# Occupational health nurses’ personal attitudes toward smoking: A cross‐sectional study

**DOI:** 10.1002/1348-9585.12221

**Published:** 2021-05-03

**Authors:** Jihye Lee, Saerom Lee, Minkyu Lee, Young Joong Kang

**Affiliations:** ^1^ Occupational Safety and Health Research Institute Korea Occupational Safety and Health Agency Ulsan Republic of Korea; ^2^ Korean Industrial Health Association Daejeon Republic of Korea; ^3^ Myoung Clinic Yeongdong‐gun Republic of Korea; ^4^ COMWEL Incheon Hospital Korea Workers' Compensation & Welfare Service Incheon Republic of Korea

**Keywords:** attitude toward smoking, occupational health nurses, occupational health service outsourcing, smoking cessation, workplace smoking cessation program

## Abstract

**Objectives:**

This study aims to investigate if experience in smoking intervention training influences attitudes toward smoking, discuss the role of health management programs of small‐ and medium‐sized enterprises, and analyze the current attitude of occupational health nurses regarding the hazards of smoking and responsibility to smokers to effectively facilitate smoking cessation support programs.

**Methods:**

We conducted an anonymous self‐administered cross‐sectional survey of 108 nurses employed in occupational health services outsourcing specialized agency in Korea. We assessed the difference in attitude about smoking according to training experience in smoking interventions and perceived competence in counseling smokers using chi‐square test and Fisher's exact test.

**Results:**

Occupational health nurses with the training experience of smoking interventions tend to perceive the harmful effects of smoking more seriously, compared to occupational health nurses without the training experience (*P* = .024, Fisher's exact test) and the OHSO nurses with the training experience tend to have professional ethics as health care professionals (*P* = .017, Fisher's exact test). Occupational health nurses having expertise in smoking cessation counseling tended to have professional ethics (*P* = .047, Fisher's exact test) and social responsibility as health care professionals (*P* = .022, Fisher's exact test).

**Conclusion:**

The occupational health nurses with training experience and expertise in smoking cessation counseling perceive the harmful effects of smoking more strongly and can enhance their professional ethics and social responsibility as health care professionals.

## 1 INTRODUCTION

Smoking is the leading cause of preventable disease, disability, and death. Worldwide, tobacco use causes more than 7 million deaths per year.^1^ Although many governments have introduced smoking bans and other effective smoking control measures to prevent smoking health burdens, cessation attempts are difficult to maintain due to tobacco's highly addictive properties. Smoking is one of the most serious global health burdens.^2^ Given that only 2% ~ 4% of smoking cessation attempts without any intervention are successful long term,^3^, ^4^ smokers need to be supported by health care professionals to achieve long‐term abstinence.^5^

The workplace, as a setting for smoking cessation research and intervention, has several advantages.^6^ Workplace smoking cessation programs appear to be more effective than clinic‐based programs in terms of the number of participants recruited and maintenance of cessation over time. In the Republic of Korea, the Ministry of Employment and Labor requires employers to manage their employees’ health in accordance with the Enforcement Decree of the Occupational Safety and Health Act. Employers must hire an occupational health manager directly or private occupational health service outsourcing (OHSO) agents. Therefore, occupational health nurses from OHSO agents regularly visit small‐ and medium‐sized enterprises (SMEs) to provide occupational health services in accordance with the Enforcement Decree of the Occupational Safety and Health Act.^7^ Under this system, if well‐trained occupational nurses regularly conduct smoking cessation counseling, they can facilitate effective smoking cessation interventions in SMEs. To our knowledge, there are few cases where SMEs conduct systematic workplace smoking cessation programs. Despite the health benefits of workplace smoking cessation programs, a lack of resources and expertise are the two main barriers to adopting health promotion programs at smaller workplaces.^8^

Previous studies have surveyed general practice in occupational health nurses in the OHSO system^7^ and investigated their perception about occupational nurses’ role^9^ under the Enforcement Decree of the Occupational Safety and Health Act in Korea. However, they have not considered the practical aspects of the occupational nurses’ tasks. This study is the first, to our knowledge, to investigate occupational health nurses’ attitudes toward the hazards of smoking and smoking cessation in the Republic of Korea, to effectively facilitate smoking cessation support programs in SMEs.

Given their critical role in the process of smoking cessation, both as advisors and behavioral models for society, previous studies have suggested that it is important to assess medical professionals’ habits and attitudes toward smoking.^10^, ^11^ These studies investigated nurses’ prevalence and personal attitudes toward smoking. However, they were mainly conducted in Western countries and with ward nurses.^11^, ^12^, ^13^ Therefore, this study focuses on occupational health nurses who provide occupational health services to SMEs and their workers in the Republic of Korea. In addition, previous studies have only examined the differences in nurses’ attitudes toward smoking concerning their smoking habits. Given that nurses are often women and there is a low smoking rate in Korean women, it is necessary to investigate factors, other than smoking prevalence, that affect nurses’ attitudes toward smoking, such as training experience in smoking cessation interventions and self‐rated expertise in smoking cessation interventions.

The OHSO system in Korea that aims to promote occupational health for SMEs can be used to manage workplace smoking cessation. Thus, the competence of the OHSO nurses would be important. This study aims to ascertain if experience in smoking intervention training influences attitudes toward smoking, discuss the role of the health management program of SMEs, and analyze the current attitude of OHSO nurses regarding the hazards of smoking and responsibility to smokers to effectively facilitate smoking cessation support programs.

## 2 MATERIALS AND METHODS

### 2.1 Study population and materials

This study involved occupational health nurses employed in a private OHSO specialized agency. In this agency, 150 nurses were involved in providing occupational health services to SMEs, with 50 to 299 employees. The OHSO nurses took over the legally imposed employers’ obligation to protect workers' health in accordance with the Enforcement Decree of the Occupational Safety and Health Act.^14^ The OHSO nurses’ duties mainly included health consultation and management in the workplaces. The work of OHSO nurses is distinct from that of nurses in wards. Detailed descriptions of OHSO programs and the work of OHSO nurses were discussed in a previous study.^7^ We contacted these 150 OHSO nurses via e‐mail and conducted a questionnaire survey from July 2019 to August 2019. The e‐mail contained a link that included a cover letter, an informed consent form, and the questionnaire. A total of 110 participants were enrolled in the study; however, two questionnaires were excluded due to inconsistent answers. Written informed consent was obtained from the participants. The study's protocol was approved by the Institutional Review Board at the Occupational Safety and Health Research Institute (approval number, OSHRI‐2019‐11).

### 2.2 Measures

The survey was conducted anonymously. The questionnaire consisted of general information, working experience, smoking status, training experience in both smoking cessation interventions and the hazards of smoking, and personal attitudes related to smoking (Appendix).

The questionnaire about general information consists of items addressing demographic characteristics, including sex, age, total working experience as a nurse, and working experience as an OHSO nurse (in years). The questionnaire of current smoking status includes the smoking record of participants, their spouses, and parents. The responses concerned their current and past smoking habits. Nurses were also asked dichotomous questions about their training experience in smoking hazards and smoking cessation interventions and about their perceived expertise to counsel smokers. Questions about personal attitudes related to smoking utilized in this study were developed and piloted by Juranić et al.^11^ We modified and translated these questions into Korean. The modification and development of the questionnaire were conducted, via the Delphi technique, by three health care professionals who worked for SAs as health managers. Each questionnaire consists of 11 questions answered on a 5‐point Likert scale, answers ranging from “strongly agree,” “agree,” “undecided,” “disagree,” to “strongly disagree.”

### 2.3 Statistical Analysis

The categorical variables were described by absolute and relative frequencies. The differences between nurses who think they have the expertise to help smokers who want to quit smoking and those who do not as well as differences between categorical variables were analyzed with a chi‐square test. If the expected frequencies were too low (more than 20% of the cells had an expected count less than 5), Fisher's exact tests were conducted instead of the chi‐square tests. The differences between nurses who had training experience in smoking cessation and those who did not as well as differences between categorical variables were analyzed with a chi‐square test. Differences in smoking intervention training experience concerning total working experience as a nurse and specific working experience as an OHSO nurse were evaluated using an independent t‐test.

The data were analyzed using SPSS version 21.0 (IBM Corp.). *P* <.05 was considered statistically significant.

## 3 RESULTS

A total of 110 nurses answered the survey, but two questionnaires had to be excluded from our analysis due to inconsistent answers. All of the 108 nurses included in the study were women. The participants’ demographic characteristics divided based on their expertise to help smokers who want to quit smoking are presented in Table 1.

**TABLE 1:** Participants' demographic characteristics divided based on expertise to help smokers who want to quit smoking (n = 108)

	Expertise^a^ (n = 47)	%	No expertise^b^ (n = 61)	%	*P* value
Age (years)					
<30	4	8.5	7	11.5	.278*
30‐39	18	38.3	30	49.2	
40‐49	13	27.7	17	27.9	
≥50	12	25.5	7	11.5	
Total experience as an OHN^c^ (years)					
mean, SD	10.22 ± 6.19		7.67 ± 5.59		**.027** **
Total experience as a nurse (years)					
mean, SD	16.61 ± 7.61		12.82 ± 6.66		**.007** **
Training experience for smoking intervention					
Yes	35	74.5	32	52.5	**.019** ***
No	12	25.5	29	47.5	
Smoking status					
non‐smokers	41	100.0	67	100.0	
Smoking status of spouse					
Current smokers	11	23.4	18	29.5	.716***
Past smokers	14	29.8	15	24.6	
Non‐smokers	16	34.0	17	27.9	
Single	6	12.8	11	18.0	
Smoking status of father					
Current smokers	17	36.2	28	45.9	.133***
Past smokers	21	44.7	16	26.2	
Non‐smokers	9	19.1	17	27.9	

^a^ Nurses who think they have the expertise to help smokers who want to quit smoking.

^b^ Nurses who do not think they have the expertise to help smokers who want to quit smoking.

^c^ Occupational health nurse.

* Fischer's exact test.

** Student *t* test.

*** Chi‐square test.

**FIGURE 1:**
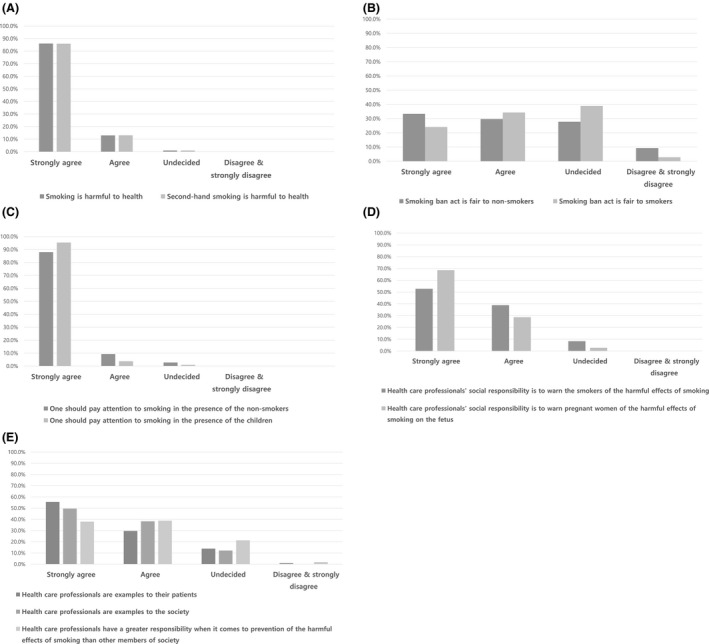
Occupational nurses’ attitude toward smoking‐related issues. (A) Attitude toward harmful effects of smoking. (B) Attitude toward smoking ban act. (C) Attitude toward smoking in front of non‐smokers and children. (D) Attitude toward health care professionals' social responsibility to warn smokers and pregnant women who smoke about harmful effects of smoking. (E) Attitude toward setting an example and responsibility of health care professionals

Figure 1 shows occupational nurses’ attitude toward smoking‐related issues. Most OHSO nurses, as health care professionals, strongly agreed on the harmfulness of smoking. They thought both direct and second‐hand smoking were harmful and that smoking in front of non‐smokers or children was inappropriate. When asked whether the anti‐smoking policy was fair to smokers/non‐smokers, there were relatively diverse answers, although there were no statistically significant differences among them. The OHSO nurses agreed strongly on the social responsibility to warn others about the harmfulness of smoking, especially pregnant women, but this answer was not statistically significant. When it comes to questions that health professionals need to be an example to others, they responded with more responsible attitudes to their patients than the unspecific counterparts, but there was no significant difference.

We examined whether training experiences in smoking cessation interventions were related to attitudes toward the harmful effects of smoking and health care professionals' social responsibility to smokers (Table 2). OHSO nurses with the training experience of smoking interventions tend to perceive the harmful effects of smoking more seriously, compared to OHSO nurses without the training experience, and the OHSO nurses with the training experience tend to have professional ethics as health care professionals.

**TABLE 2:** Relationship between the training experience in smoking cessation and the perceived harmfulness of smoking and social responsibility of health care professionals

	Training experience^a^ (n = 67)		No training experience^b^ (n = 41)		Total		*P* value*
	n	%	n	%	n	%	
Smoking is harmful to health							
Strongly agree	62	92.5%	31	75.6%	93	86.1%	**.024**
Agree	5	7.5%	9	22.0%	14	13.0%	
Undecided	0	0.0%	1	2.4%	1	0.9%	
Disagree & strongly disagree	0	0.0%	0	0.0%	0	0.0%	
Health care professionals have a social responsibility to warn smokers of the harmful effects of smoking.							
Strongly agree	42	62.7%	15	36.6%	57	52.8%	**.017**
Agree	22	32.8%	20	48.8%	42	38.9%	
Undecided	3	4.5%	6	14.6%	9	8.3%	
Disagree & strongly disagree	0	0.0%	0	0.0%	0	0.0%	

^a^ Nurses with training experience in interventions to stop smoking.

^b^ Nurses without training experience in interventions to stop smoking.

* Fischer's exact test.

Table 3 shows whether having expertise in smoking cessation interventions is related to health care professionals’ attitudes toward their social responsibility to warn others about the harmful effects of smoking. In addition, whether this expertise provides them with increased social responsibility to prevent the harmful effects of smoking compared to other members of society. OHSO nurses having expertise in smoking cessation counseling tend to have professional ethics and social responsibility as health care professionals.

**TABLE 3:** Relationship between the perceived expertise in smoking cessation and the social responsibility of health care professionals

	Expertise^a^ (n = 47)		No expertise^b^ (n = 61)		Total		*P* value*
	n	%	n	%	n	%	
Health care professionals' social responsibility is to warn smokers of the harmful effects of smoking							
Strongly agree	31	66.0%	26	42.6%	57	52.8%	**.047**
Agree	14	29.8%	28	45.9%	42	38.9%	
Undecided	2	4.3%	7	11.5%	9	8.3%	
Disagree & strongly disagree	0	0.0%	0	0.0%	0	0.0%	
Health care professionals have a greater responsibility than other members of society when it comes to prevention of the harmful effects of smoking							
Strongly agree	25	31.1%	16	46.8%	41	38.0%	**.022**
Agree	13	39.3%	29	38.3%	42	38.9%	
Undecided	8	27.9%	15	12.8%	23	21.3%	
Disagree & strongly disagree	1	2.1%	1	1.6%	2	1.9%	

^a^ Nurses who think they have the expertise to help smokers who want to quit smoking.

^b^ Nurses who do not think they have the expertise to help smokers who want to quit smoking.

* Fischer's exact test.

## 4 DISCUSSION

We investigated OHSO nurses’ personal attitudes toward smoking, associated factors influencing their attitude toward the hazards of smoking, and their role in the process of smoking cessation both as advisors and behavioral models. Furthermore, we compared attitudes based on experience in smoking intervention training and personal opinions about one's expertise to help smokers quit smoking. The result of this study revealed that OHSO nurses with training experience and who have expertise in smoking cessation counseling take the harmful effects of smoking more seriously; also, their training and expertise enhance their professional ethics and social responsibility as a health care professional.

The results of this study suggest that it is necessary to provide nurses with adequate opportunities to learn about smoking cessation intervention techniques, the hazards of smoking, and their role as both smoking cessation advisors and behavioral models for society. Previous studies that investigated nurses’ attitudes toward smoking have suggested the necessity of developing appropriate training programs to improve nurses’ ability to provide active support to smokers in smoking cessation interventions.^12^, ^13^ Juranić et al. investigated the prevalence, habits, and personal attitudes toward smoking among nurses and highlighted the necessity of providing appropriate training programs to improve their ability in smoking cessation techniques.^11^ The workplace appears to be a useful setting to help people quit smoking. Large groups of smokers can be easily reached and helped by proven methods if a workplace has an adequate professional anti‐smoking program.^15^ In Korea, the OHSO system, which promotes occupational health in SMEs, can be used to manage workplace smoking cessation. Thus, the competence of the OHSO nurses is important. Experience in smoking intervention training can help health care professionals become more active and confident in promoting smoking cessation.

Although previous studies suggested that health professionals’ smoking behavior impacts their attitude toward smoking,^11^, ^12^, ^16^ we could not analyze the association between the participants’ smoking behavior and attitudes toward smoking since all of the participants of our study had never smoked. Therefore, we analyzed whether the participants’ spouse or parents smoked and found that family members’ smoking habits did not have a significant impact on nurses’ attitude toward smoking (data not shown). Willaing et al.^12^ revealed that the smoking behavior of health professionals affects attitudes toward smoking, and the lack of self‐rated qualification as a smoking cessation advisor is an important barrier to being a smoking cessation advisor. The less qualified the health care professionals feel, the less they counsel.

Despite the evidence that many smokers want to and have tried to quit smoking, it is very rare to maintain long‐term abstinence from smoking.^3^ Tobacco dependence has been recognized as a chronic condition that needs repeated long‐term interventions.^17^ Professional support in the workplace provides an opportunity to quit smoking even for those who do not intend to.^18^ The involvement of health professionals in tobacco cessation efforts at all levels is necessary to facilitate smoking cessation. Despite the health benefits of workplace smoking cessation programs, there are some barriers to implementing or sustaining workplace health management programs, including a lack of resources, expertise, and employee interest.^8^, ^19^

In the Republic of Korea, employers must hire an occupational health manager directly or hire private OHSO agents. The OHSO nurses in the Republic of Korea regularly visit SMEs with 50 to 299 employees to provide occupational health services, primarily health consultations,^7^ in accordance with the Enforcement Decree of the Occupational Safety and Health Act.^14^ Therefore, OHSO nurses can approach the current health status of employees in a relatively specific way and conduct more complex counseling and health management. This will lead to a more efficient intervention to help workers quit smoking. If workplaces take advantage of OHSO nurses’ regular visits and have systematic smoking cessation interventions, these interventions can be the basis for more advanced strategies. The worksites can provide sustained peer group support and positive peer influence for smoking cessation and provide access to a large number of smokers with or without smoking‐related diseases.^6^ Recent research indicates that more complex approaches, combining two or more different elements for anti‐smoking intervention, can achieve better success rates for smoking cessation.^20^ The OHSO nurses could be the main conductors and counselors of workplace smoking cessation programs and encourage smokers to join the nationwide smoking cessation support program conducted by the NHIS in the Republic of Korea.^21^

The nurses with low self‐rated qualification and low expertise would likely be passive in referring smokers to smoking cessation clinics and fail to provide smokers proper smoking cessation information and aids.^12^ Previous studies commonly emphasize the necessity of proper education to help health professionals take responsibility for counseling smokers about smoking hazards and smokers’ health. Although there were no smokers among the nurses in our study, we were able to identify other factors influencing attitudes toward smoking based on whether they had training experience of smoking and expertise to counsel smokers.

OHSO nurses answered that the smoking ban act is fair to non‐smokers, but they are often unsure whether the act is fair to smokers. This can be due to a lack of awareness that smoking regulation can also help smokers’ health. OHSO nurses responded that smoking in the presence of children is more inappropriate than smoking in the presence of non‐smokers and that they had a more ethical and social responsibility to inform pregnant women of the harmful effects of smoking than other smokers.

When asked whether health professionals should set an example for smokers, most of the OHSO nurses strongly agreed that they should be an example for their patients, but were more passive when asked whether they should be an example to society or have a greater responsibility than other members of society when it comes to prevention of the harmful effects of smoking. This is similar to the results of a previous study on health care professionals.^11^ Most nurses agree with the harmfulness of smoking or the role of medical personnel, but there are differences in their degree of agreement. OHSO nurses would be relatively passive in encouraging ordinary, healthy workers to quit smoking when they counsel them at the workplace. This attitude might be due to considering smoking as more harmful to patients and pregnant women or as a personal preference rather than a health hazard to healthy workers. OHSO nurses need to be encouraged to have a responsible attitude and the confidence to actively conduct anti‐smoking programs. Appropriate education about smoking cessation and a competent attitude toward smoking cessation counseling by health professionals can be an effective way to successfully prevent smoking‐related health risks.

To our knowledge, this is the first study that reported OHSO nurses’ attitudes toward smoking and experience and confidence in smoking cessation interventions. As a result of the study, the authors can emphasize the importance of education in OHSO nurses, to promote the SMEs’ workplace health management under the OHSO system, and to facilitate the national smoking cessation program of the NIH. Despite its strengths, our study is subject to certain limitations. First, this study is cross‐sectional and can only reveal limited relationships between training experience in smoking cessation interventions and attitude toward smoking; it cannot determine the temporal relationships. Our results cannot establish a causal link between training experience and OHSO nurses’ attitude to smoking and related factors. Second, we modified and translated the questionnaire of Juranić et al.; however, the questionnaire was not validated during the modification. Although there are limitations compared to previous studies, we believe that our questionnaire was appropriate for investigating the perspective and attitudes of OHSO nurses in Korea. Third, we showed selection bias in recruiting respondents from a private OHSO agency. This study is the first investigating the work attitude of occupational health nurses in the Republic of Korea, so further research is needed to generalize our findings. Fourth, there are subjective measures in the questionnaire. Training experience in smoking cessation interventions was revealed only through respondents’ self‐report; the characteristics of training, such as level, duration, and efficiency, were not revealed. Self‐reported expertise is also a subjective measure, as it is difficult to objectively measure expertise. However, a previous study suggested that the lack of self‐rated qualification is a barrier to being an active smoking intervention advisor. This uncertainty could potentially render the result less reliable. Further research is needed on what kind of education would foster OHSO nurses’ responsible attitude and high professional ethics about smoking and occupational health to resolve this uncertainty. Although well‐trained OHSO nurses are not a guaranteed method to achieve workplace smoking cessation management, it would be worthwhile to explore various approaches to facilitate the workplace smoking cessation program. Indeed, it has been suggested that combining two or more different elements for anti‐smoking intervention can achieve better success rates for smoking cessation.^20^

Despite the study's limitations, we emphasize the importance of our findings regarding OHSO nurses’ competence and attitude related to their work because the OHSO system is, in addition to workers’ health examination, a major occupational health management system for SMEs in accordance with the Enforcement Decree of the Occupational Safety and Health Act of Korea. In addition, despite the important role of the system, no research has been conducted to improve the work ability of OHSO nurses, one of the main health personnel of this system. Efficient use of the OHSO system and nurses would enable the implementation of a complex approach for workplace smoking cessation.

## 5 CONCLUSION

This study investigated OHSO nurses’ attitudes toward smoking and their training experience and competence in smoking cessation interventions to discuss the necessity for additional education. Having well‐trained OHSO nurses does not always guarantee the success of workplace health management and smoking cessation programs. However, the OHSO system with well‐trained nurses can contribute to SMEs’ workplace health management and facilitate the nationwide smoking cessation program.

## AUTHOR CONTRIBUTIONS

Conceptualization, KYJ, LJH, and LSR; methodology, KYJ; validation, LSR; formal analysis, LJH; investigation, LJH and LSR; resources, LSR; data curation, KYJ and LJH; writing‐original draft preparation, KYJ, LJH, and LSR; writing‐review and editing, KYJ, LJH, LMK, and LSR; visualization, LJH; supervision, KYJ; project administration, KYJ; All authors have read and agreed to the published version of the manuscript.

## DISCLOSURES

*Approval of the research protocol:* This study was approved by the Institutional Review Board at the Occupational Safety and Health Research Institute (approval number, OSHRI‐2019‐11) and conformed to the ethical principles for research involving human participants outlined in the Declaration of Helsinki. *Informed Consent:* Participants provided written informed consent. *Conflict of Interest:* None declared.

## Supporting information

Supplementary Material
