# Supplementary material for: Occupational health nurses’ personal attitudes toward smoking: A cross‐sectional study
Source: J Occup Health. 2021 May 3;63(1):e12221. doi: 10.1002/1348-9585.12221 (PMC8090975; doi:10.1002/1348-9585.12221)
Supplement: Supplementary file 1 — Supplementary Material [file JOH2-63-e12221-s001.docx]

**Appendix**

**Demographic characteristics**

1. Sex

a) Male b) Female

2. Age

( )

3. How many years have you worked at the current institution? (Not shown in the table)

( )

4. How many years have you worked as an occupational health nurse?

( )

5. What is your total experience as a nurse?

( )

6. Are you currently a smoker?

a) Yes

b) No, I have never smoked.

c) Past smoker, no smoking since ( ) years ago

7. Is your spouse a smoker?

a) Yes

b) No, he/she has never smoked.

c) Past smoker, no smoking since ( ) years ago

d) I am single

8. Is your father a smoker?

a) Yes

b) No, he has never smoked.

c) Past smoker, no smoking since ( ) years ago

**Questionnaire for the attitude toward smoking.**

*Attitudes toward the harmful effects of smoking

1. Smoking is harmful to health

a) Strongly agree

b) Agree

c) Undecided

d) Disagree

e) Strongly disagree

2. Second-hand smoking is harmful to health

a) Strongly agree

b) Agree

c) Undecided

d) Disagree

e) Strongly disagree

*Attitudes toward the Smoking Ban Act

3. The Smoking Ban Act is fair to non-smokers

a) Strongly agree

b) Agree

c) Undecided

d) Disagree

e) Strongly disagree

4. The Smoking Ban Act is fair to smokers

a) Strongly agree

b) Agree

c) Undecided

d) Disagree

e) Strongly disagree

*Attitudes toward smoking in front of non-smokers and children

5. One should pay attention to smoking in presence of non-smokers

a) Strongly agree

b) Agree

c) Undecided

d) Disagree

e) Strongly disagree

6. One should pay attention to smoking in presence of children

a) Strongly agree

b) Agree

c) Undecided

d) Disagree

e) Strongly disagree

*Attitudes toward health care professionals’ ethical responsibility to warn smokers and pregnant women who smoke about the harmful effects of smoking

7. The health care professionals’ ethical responsibility is to warn smokers of the harmful effects of smoking

a) Strongly agree

b) Agree

c) Undecided

d) Disagree

e) Strongly disagree

8. The health care professionals’ ethical responsibility is to warn pregnant women of the harmful effects that smoking has on the fetus

a) Strongly agree

b) Agree

c) Undecided

d) Disagree

e) Strongly disagree

*Attitudes toward the example and responsibility of health care professionals

9. Health care professionals are examples to their patients

a) Strongly agree

b) Agree

c) Undecided

d) Disagree

e) Strongly disagree

10. Health care professionals are examples to society

a) Strongly agree

b) Agree

c) Undecided

d) Disagree

e) Strongly disagree

11. Health care professionals have a greater responsibility when it comes to the prevention of the harmful effects of smoking than the other members of society

a) Strongly agree

b) Agree

c) Undecided

d) Disagree

e) Strongly disagree

**Dichotomous questions.**

1. Have you ever received any specialized training in smoking hazards and smoking cessation treatment?

a) Yes b) No

2. Do you think you have the level of expertise to help smokers who want to quit smoking?

a) Yes b) No
